# Little Men

**Published:** 1888-11-10

**Authors:** 


### Little Men.

Every position has its own peculiar opportunities for the gaining of knowledge and understanding of human life. The family doctor thinks he finds his way deeply into the secrets of personal experience, and so he does. But the newspaper editor who shows himself by his writings to have a deep sympathy with the varying circumstances of his readers has opportunities of another kind, opportunities which are altogether unique. The editor is constantly under the temptation to make interesting "copy." But we venture to think that no position demands greater self-restraint and consideration for others than does his. Interesting copy he may, and ought to make; but always legitimately, and always in such a way as to prove himself worthy of the wonderful variety of confidences reposed in him.

This brief preface is called forth by a peculiar letter received a short time ago, from "Parvus." "Parvus," as his name implies," is a little man, or perhaps he ought rather to be called a little boy, since he is only eighteen years of age. He is but five feet one in his stockings. He puts two questions, which because of their simplicity and evident sincerity we print in full: "Do you think," is his first question, "that by a selected diet it is possible to increase one's height, provided the growing age has not passed?" "Do you think," he says secondly, "it is likely I shall grow any more? I am nearly nineteen, and only 5 feet 1 inch in my stockings. Can you suggest anything likely to make me grow?"

The first answer we have to make is that we have two standing rules, both of which bear somewhat upon the present case. One is, never to advise any kind of medical treatment; the other, never to name any particular medical man to inquirers. These rules are found to be quite necessary, and we never depart from them. We cannot, therefore, in this instance con sider the questions medically, but only from a kind of scientific and humanitarian point of view.

It is in many respects a misfortune to be unduly short, as it is to be unduly tall. The very little boy at school, for example, is often bullied by the big boy. At cricket, and still more at football, the short fellow, especially if he be also slim, is generally at a discount. When the beard begins to sprout, and the Eton jackets are exchanged for frock coats or evening dress, brevity of the legs and a corresponding condition of the body insist upon putting themselves in evidence. If a young man of five feet one or two persuades himself that in a swallow-tail and high-heeled boots he is really fairly tall, he soon discovers his mistake when he enters Mrs. Smith's dancing-room. Most of the young ladies have their programmes full when he asks for a dance, and he often finds that he must console himself with the delights of meditation as a "wallflower." When he seeks an introduction to business, or decides for the church, medicine, or the bar, he finds at the outset that his want of stature corresponds in a certain limited sense to want of capital. A tall man with a "good presence" has undoubtedly, at the commencement, a decided advantage over his fellow whose appearance is less pronounced and effective.

But—and there is a whole world of virtue in this particular "but"—no man ever spoke a truer or profounder word in his life than Dr. Isaac Watts, when he said—

"The mind's the standard of the man."

All the disadvantages and disabilities of short stature go down and disappear one by one before courage, conduct, and sense. Even at school the little boy's pluck often keeps the big bully on terms of respectful neutrality. In the drawing-room, the short man who can sing and dance and behave himself like a gentleman soon acquires a popularity which the six-foot lout strives for in vain. At business his promptitude and attention—and in one of the professions his brains and character—very quickly tell their own story, and win their own battles. Everywhere the short man of real capacity finds that niggard nature has done him no lasting injury, but that in spite of her sulkiness in leaving him like a palmtree with a stunted trunk, his pluck and determination have enabled him to reach mountain heights, whence he can look down on those whose physical stature exceeds his own. Lest any reader should think that there is some special pleading here, the writer ventures to explain that he only wants an inch and a-half of six feet.

As to the actual questions which "Parvus" has put before us, and we before our readers, there is really no need for despair. Many a boy and girl of eighteen is still in the growing stage, and sometimes for long after that period. Much depends on circumstances. Is an outdoor life possible? Has "Parvus" the opportunity and the capacity for plenty of vigorous exercise? Is he of temperate, chaste, and healthy habits? These circumstances all have an important bearing on the facts at issue. Perhaps the most important question of all is that of heredity. Are the parents tall? Or is one of them above the middle size? Or if the immediate progenitors are not tall, were the grandparents, on either side or both, of more than average height? If there is a decided history of family tallness on either or both sides, undoubtedly "Parvus" may expect that, in spite of his age, which, after all, is not so very advanced, he may yet reach middle or more than middle height.

Some persons may smile at the question asked with regard to selected diet. But it is not a foolish question at all. "Parvus" probably knows, as does every man who has had to do with the breeding and rearing of stock, that diet exercises a profound influence, not merely on the fattening, but also on the increasing of the size of animals of all kinds. Horses, cattle, sheep, pigs, fowls, dogs, and all other domestic animals may be gradually increased in size to a very marked degree. It must, of course, be remembered that this process generally requires to be extended over several generations. Still each generation contributes its quota of increase. There can be no doubt whatever that if intelligent physicians could exercise the same control over human creatures that stock rearers do over their animals a race very much improved, both physically and mentally, would be produced in a few generations. It is entirely within the right of a scientific age that it should demand from its physicians the clear enunciation and constant enforcement of such simple general principles as will tend in the direction of physical and mental development and progress. A medical profession which merely treats disease is altogether unworthy of a scientific and progressive epoch. Medicine must both teach and lead. It must teach and lead not only the select few who devote themselves to its practice, but the whole world of which it is a part, and to which it owes the common indebtedness that the whole has a right to claim from each of its parts. It is a thesis which cannot be too often or too strenuously maintained that "medicine was made for man, not man for medicine." The average man looks upon the world too much as his oyster, which he must steadfastly endeavour to open in his own peculiar interests. That is but one point of view, and a very inadequate one if alone persisted in. However much the world may owe to medicine, or any other single profession or calling, it must always be true that medicine owes still more to the world. The whole is always, and in every sense, greater than the part.

